# Case Report: Effective use of omadacycline in Japanese spotted fever

**DOI:** 10.3389/fmed.2026.1882022

**Published:** 2026-07-08

**Authors:** Xue Dai, Jia-Ling Bu, Yuan Peng, Wang Deng

**Affiliations:** 1 Department of Respiratory and Critical Care Medicine, Second Affiliated Hospital of Chongqing Medical University, Chongqing, China; 2 Key Laboratory of Respiratory Inflammatory Injury and Precision Diagnosis and Treatment, Chongqing Municipal Health and Health Committee, Chongqing, China

**Keywords:** infectious disease, Japanese spotted fever, lung, MODS, omadacycline

## Abstract

This case report describes the efficacy of omadacycline for the treatment of Japanese spotted fever. A 57-years-old woman presented with fever and maculopapular rash, followed by dyspnea. Laboratory abnormalities included respiratory failure, thrombocytopenia, liver dysfunction, and elevated inflammatory markers. *Rickettsia japonica*, the causative pathogen, was detected in bronchoalveolar lavage fluid (BALF). Based on the clinical manifestations, laboratory findings, and microbiological evidence, a diagnosis of Japanese spotted fever caused by *Rickettsia japonica* was considered. She received anti-infective therapy, intravenous polyene phosphatidylcholine, human albumin and platelet transfusions, along with symptomatic supportive care. After 14 days of omadacycline treatment, inflammatory markers and platelet counts normalized, and chest CT showed significant improvement, leading to full recovery and discharge. This case indicates that omadacycline may have contributed to the patient’s recovery, alongside multiple concurrent treatments.

## Introduction

Japanese spotted fever is a rickettsial disease caused by *Rickettsia japonica*, first reported in Japan (1). The incidence of this disease has shown an increasing trend in China in recent years (2, 3). The main clinical manifestations are fever and maculopapular rash, while DIC or abnormal liver function may occur in fatal cases (4). This case report illustrates the clinical application of omadacycline in a patient with severe Japanese spotted fever complicated with multiple organ failure. The drug may have potential value for the clinical treatment of such cases.

## Case report

The patient, a 57-years-old Chinese woman from Chongqing, frequently visited mountain forests for cooling during the past 3 months. One week prior to admission, the patient presented with fever, chills, and rigors, with a maximum temperature of 39.2 °C. After receiving intravenous fluid therapy at a local clinic (intravenous ceftriaxone combined with levofloxacin), her symptoms showed no significant improvement. Subsequently, a scattered maculopapular rash emerged on the trunk and extremities the patient was admitted to a county-level hospital in Chongqing. Despite anti-infective and symptomatic treatment, there was no significant improvement. Additionally, blood tests showed markedly decreased platelets (34 × 10^9^/L), prompting transfer to our hospital.

On admission, the patient presented with fatigue, dyspnea and bilateral lung rales. Physical examination revealed a generalized non-pruritic erythematous maculopapular rash diffusely involving the face, trunk and extremities. No vesicles, ecchymoses or pathognomonic eschar were identified. Arterial blood gas analysis under nasal cannula oxygen showed a PO2 of 46.0 mmHg. These findings suggest possible multi-organ dysfunction. Vital signs (BP 146/98 mmHg, P 113 bpm, R 24/min, Temp 37.1 °C) and comprehensive physical examination including the neurological and cardiovascular systems showed no remarkable abnormalities. The patient’s disease severity scores at admission were as follows: APACHE II, 27; SOFA, 12; GENEVA, 5.

Laboratory findings at admission revealed systemic abnormalities indicative of inflammation and multiple organ dysfunction. On admission, laboratory tests showed: WBC 13.38 × 10^9^/L, Neutrophil count 12.81 × 10^9^/L, CRP 180.84 mg/L, IL-6 592.92 pg/mL [normal range, (0.0−5.3)], IL-10 524.15 pg/mL [normal range, (0.0−4.91)], IFN-γ 422.31 pg/mL [normal range, (0.0−7.42)], PCT 0.49 ng/mL, HBP 37.43 ng/mL, indicating active inflammation; arterial blood gas analysis showed pH 7.390, PaO2 46.0 mmHg, consistent with type I respiratory failure; PLT 19.0 × 10^9^/L, indicating high bleeding risk; D-dimer > 10,000.0 ng/mL [normal range, (0.0–550.0)], coagulation: APTT 46.2 s [normal range, (28.0−44.0)], APTT-R 1.40 [normal range, (0.8−1.3)], FDP 61.91 μg/mL [normal range, (0.01−5.0)], suggesting coagulopathy with severe infection; electrolytes: Na^+^ 132.2 mmol/L [normal range, (137.0−147.0)], Ca^2+^ 1.82 mmol/L [normal range, (2.12−2.92)], indicating imbalance; liver function tests revealed ALT 62 U/L, AST 185 U/L, TBil 30.2 μmol/L, Alb 25.8 g/L, LDH 723 U/L [normal range, (120.0−250.0)]. The patient had impaired liver function and hypoalbuminemia, likely due to multi-organ dysfunction induced by inflammation.

Bronchoalveolar lavage fluid metagenomic DNA sequencing revealed 3 reads of *Rickettsia japonica* and 394 reads of *Mycobacterium tuberculosis* complex (sputum acid-fast bacilli smear was negative). Post-treatment laboratory indices returned to near-normal levels (Table 1).

**TABLE 1:** Laboratory test results.

Test items	Result (admission)	Result (after treatment)	Reference range
WBC (×10^9^/L)	13.38	8.66	3.5–9.5
Neutrophil count (×10^9^/L)	12.81	7.09	1.80–6.30
CRP (mg/L)	180.84	19.95	0.0–10.0
PLT (×10^9^/L)	19.0	471.0	100.0–300.0
RBC (×10^12^/L)	4.12	3.05	3.8–5.1
Hb (g/L)	129	94	115.0–150.0
ALT (U/L)	62.0	52	7.0–40.0
AST (U/L)	185.0	41	13.0–35.0
Alb (g/L)	25.8	32.5	40.0–55.0
TBil (μ mol/L)	30.2	13.6	2.0–20.0
PCT (ng/ml)	0.49	<0.05	0.0–0.05
HBP (ng/ml)	37.43	18.72	0.0–15.0
PO_2_ (mmHg)	46.0	149	60.0–100.0
PCO_2_ (mmHg)	39.0	30	35.0–48.0

After treatment, laboratory indices generally returned to normal. The patient received multiple platelet transfusions for severe thrombocytopenia at admission, which together with rebound thrombocytosis after infection control led to a marked rise in platelet count. The drop in hemoglobin resulted from systemic consumption by acute infection and repeated blood sampling.

Imaging findings: CT showed with small-to-moderate bilateral pleural effusions causing partial compression atelectasis of the lower lobes and scattered pulmonary inflammation with possible interstitial pulmonary edema. These findings indicate severe pulmonary infection with pleural effusions (Figure 1).

**FIGURE 1:**
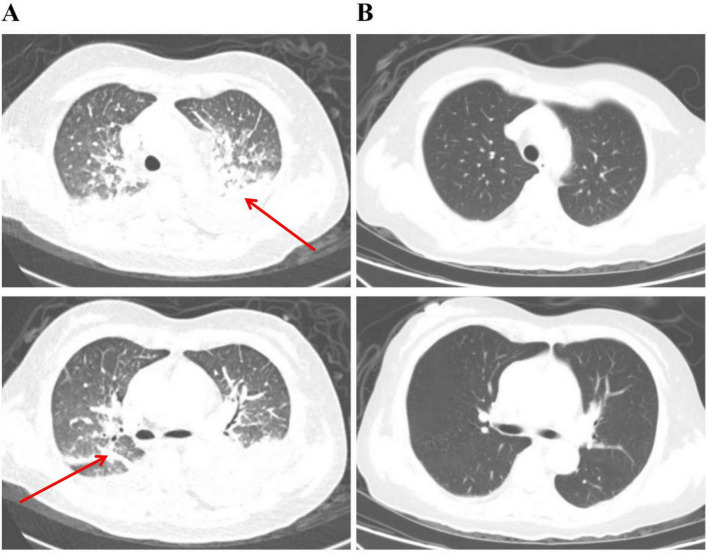
Chest CT images: **(A)** Baseline (at admission); **(B)** After 2 weeks of treatment. On admission: Mild to moderate bilateral pleural effusion was noted, with partial atelectasis of the bilateral lower lung lobes. Patchy opacities were scattered in both lungs, and interlobular septal thickening was also observed bilaterally. Two weeks after treatment: Only a few faint patchy opacities remained in both lungs. The bilateral lower lobes were fully re-expanded. No hilar or mediastinal lymphadenopathy was detected. There was no pleural thickening or residual pleural effusion on either side.

Considering the patient’s outdoor exposure history, generalized maculopapular rash, laboratory and imaging evidence of severe infection complicated by coagulopathy and multiple organ failure, and low reads of *Rickettsia japonica* identified by mNGS in BALF, we made a definitive diagnosis of rickettsial infection. Despite the low mNGS reads, the diagnosis was confirmed comprehensively by clinical manifestations, imaging findings and favorable response to targeted treatment. This diagnosis guided the subsequent treatment, which proved effective.

Upon admission, the patient was admitted to the RICU with respiratory failure and received non-invasive ventilation. Initial empirical meropenem treatment failed to relieve persistent fever, and switching to imipenem/cilastatin combined with teicoplanin still yielded poor fever control. On hospital day 3, based on BALF mNGS pathogen results, all previous antibacterial agents were discontinued. The regimen was switched to intravenous omadacycline for anti-rickettsial therapy, combined with isoniazid and ethambutol for anti-tuberculosis treatment. Shortly after the regimen adjustment, the patient’s fever resolved and platelet count gradually recovered. The anti-tuberculosis drugs were discontinued immediately after fever relief, and no disease recurrence was observed. Omadacycline was administered for 2 weeks, followed by 1 week of oral minocycline sequential therapy. The patient achieved complete clinical recovery and was discharged. Notably, multiple concurrent medications and supportive interventions were given throughout hospitalization, which may have contributed to the overall clinical improvement.

## Discussion

In this study, we report a case of omadacycline-treated Japanese spotted fever with respiratory failure. Rickettsiae are primarily transmitted through arthropod bites, causing diseases of varying severity (5). Cases have been reported in Hubei (6), Zhejiang (7), and Anhui (8) provinces of China. The typical manifestations of Japanese spotted fever include high fever, chills, and diffuse erythema (9, 10). Laboratory findings revealed leukocytosis, thrombocytopenia, elevated CRP, and increased hepatic transaminases (11). This report is an exploratory clinical observation, and more clinical data are required to confirm its general applicability.

The infection may lead to severe complications such as pneumonia, coagulopathy, disseminated intravascular coagulation and multiple organ dysfunction syndrome. Untreated, this could progress to DIC and multiple organ dysfunction syndrome (MODS) (12). This disease shares similar clinical manifestations with various other conditions, and delayed or ineffective diagnosis and treatment may increase patient mortality. Therefore, early diagnosis and management remain a clinical challenge.

The critically ill patient received anti-infective therapy to combat the *Rickettsia japonica* infection and prevent disease progression. This was supplemented with supportive care, including bronchoalveolar lavage and infusions of polyene phosphatidylcholine, recombinant human platelets, human albumin, and immunoglobulin, to maintain organ function and stabilize the patient’s condition. Tetracyclines are the standard treatment for rickettsial infections. Omadacycline, a semi-synthetic derivative of minocycline, has strong antimicrobial potency and a broad spectrum of activity, and may help address drug resistance (13). Omadacycline has demonstrated potential efficacy in treating rickettsial infections, as previously reported (14, 15). Additionally, omadacycline exhibits minimal hepatic effects, eliminating the need for dose adjustment in patients with impaired liver function. However, the evidence regarding the use of omadacycline in rickettsial infection-induced MODS remains limited. In this case involving multiple treatments, omadacycline may have played a supportive role in disease control. It should be noted that the patient received sequential antibiotics, anti-tuberculosis agents, liver-protective drugs, blood product transfusions and comprehensive supportive care during hospitalization. These multiple concurrent interventions introduced significant confounding factors. Accordingly, we cannot attribute the patient’s recovery solely to omadacycline, and this case only serves as a clinical experience rather than evidence to verify the independent efficacy of the drug.

This case highlights the complexity of diagnosing and treating severe Japanese spotted fever, particularly the need for prompt differential diagnosis when MODS is present. Compared with previously published literature, our report further complements the clinical experience of using omadacycline in severe rickettsial infection accompanied by MODS. Early diagnosis and treatment are crucial for reducing rickettsial infection mortality, while supportive care is equally important.

## Data Availability

The raw data supporting the conclusions of this article will be made available by the authors, without undue reservation.
